# Research Progress on Rice-Blast-Resistance-Related Genes

**DOI:** 10.3390/plants14172698

**Published:** 2025-08-29

**Authors:** Biaobiao Cheng, Beibei Lv, Qiangbing Xuan, Yunfang Li, Jing Li, Weihong Liang, Junjie Wang

**Affiliations:** 1 College of Life Science, Henan Normal University, Xinxiang 453007, China; cbb521970@outlook.com (B.C.);; 2 The Observation and Research Field Station of Taihang Mountain Forest Ecosystems of Henan Province, Xinxiang 453007, China

**Keywords:** rice, rice blast, resistance gene, susceptibility genes, breeding

## Abstract

As a staple food crop, *Oryza sativa* L. is not only the basis of global food and nutrition security but also an important cornerstone of national economic development and social stability. However, the growth of rice is often accompanied by the threat of rice blast, which can lead to the death of seedlings or plants before heading. In the later stages of growth, a severe leaf blast infection will reduce the leaf area at the filling stage, thereby reducing the grain yield. The study of rice blast resistance genes and susceptibility genes is a key strategy for controlling the occurrence of rice blast and ensuring sustainable rice production. This paper reviews the impact of rice blast on the global economy and food security in recent years, describes the immune mechanism of rice blast resistance, and introduces the latest progress in related research. At the same time, the main genes of rice blast resistance and the resistance-related genes, as well as the susceptibility genes identified or cloned in recent years, are summarized. This paper also discusses the application of conventional breeding, molecular-marker-assisted breeding, gene editing, and other technologies in rice blast resistance breeding. The problem of accurately finding avirulence genes for *R* genes in current disease-resistant breeding is discussed and explored, aiming to improve rice blast resistance, agronomic traits, and yield in a sustainable way.

## 1. Introduction

*Oryza sativa* L. cultivation worldwide accounts for about 140 million to 157 million hectares, accounting for 90% of the total planting area, as about 50% of the population (mainly in Asia and Africa) relies on it as a staple food, meaning that rice plays an important role in food security [1]. According to the Food and Agriculture Organization (FAO) [2], global food production needs to increase by about 60% by 2050 to meet the needs of the rapidly growing population. However, rice blast is a common disease in the rice-planting process, which reduces global rice production by about 30% every year, and the yield loss can be as high as 50%, posing a serious threat to food security [3]. *R* genes are important components of rice’s immune system, specifically recognizing pathogens and activating immune responses (including triggering pattern immunity, triggering immunity, or atypical immune pathways) that confer resistance to rice blast [4]. In this paper, the immune mechanism of rice against rice blast is systematically reviewed, and the resistance-related genes and susceptibility genes, including the main genes, protein kinases, and transcription factors involved in rice’s resistance to rice blast, are summarized. At the same time, the application of conventional breeding, molecular marker-assisted breeding, gene editing, genomics-assisted breeding, rapid breeding, and artificial intelligence combined with these new genomics and biotechnology methods in rice blast resistance breeding is discussed.

## 2. Molecular Mechanisms of Rice Immunity and Rice Blast Invasion

The survival of rice depends on its innate immune system, which consists of two layers: pathogen-associated molecular pattern (PAMP)-triggered immunity (PTI), and effect-triggered immunity (ETI). PTI is triggered by cell-surface-localized pattern-recognition receptors (PRRs), while ETI is activated by pathogen effector proteins through intracellular localized receptors called nucleotide-binding domain leucine-rich repeats (NLRs) [5]. Once the rice is infected with pathogenic bacteria, the two immunizations of PTI and ETI will be gradually activated to resist the systematic invasion of rice blast. Therefore, in-depth study of plant resistance mechanisms is the key to effective prevention of rice blast.

### 2.1. Infection Mechanism of Rice Blast Fungus

*Magnaporthe oryzae* is internationally recognized as the most harmful fungal pathogen with respect to rice; it belongs to the Ascomycota, and it has two types: asexual and sexual, belonging to Botrytis and Ascomycota, respectively [6]. The asexual rice blast fungus mainly uses conidia and hyphae as the source of infection; it has the characteristics of strong infection ability and rapid spread, which can greatly reduce the yield of rice. In severe cases, it is accompanied by no harvest of rice throughout the year [7]. The disease can be divided into four types—seedling blast, leaf blast, panicle blast, and joint blast—according to the different occurrence periods and the different parts damaged by the disease [8].

**Figure 1:**
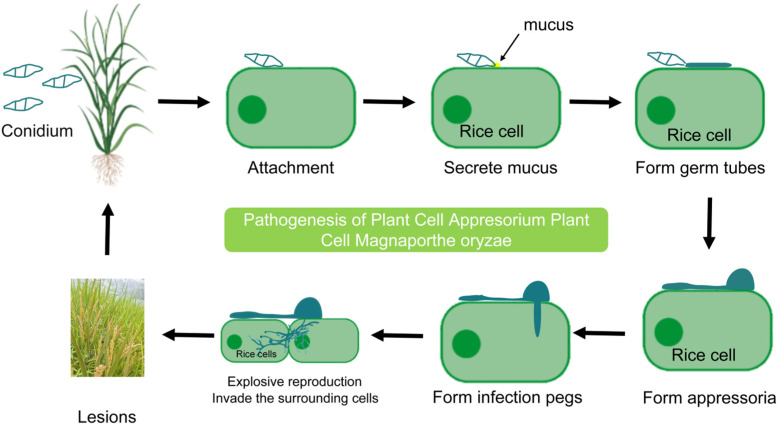
Mechanism of rice blast infection: This figure shows the process of rice blast infection. Conidia adhere to rice leaves through the medium and secrete mucus to form penetrating hyphae, which gradually evolve into spherical infectious hyphae and secrete effector proteins. Conidia invade adjacent cells through plasmodesmata, thereby damaging tissues and forming lesions. The lesions produce conidia again to initiate a new round of infection.

The conidia of rice blast fungus are transmitted through various media, form dome-shaped appendages after they are born on the surface of rice, and secrete mucus to firmly adhere to the leaf surface in order to invade plant tissues. The attachment continuously absorbs water from the leaf dew and accumulates osmotic fluid. As repolarization progresses, the top of the attachment will produce puncture nails, which will destroy the host cell wall with the help of mechanical force [9]. Subsequently, the penetration hyphae differentiate into bulbous invasive hyphae at the host plasma membrane and secrete effector proteins, which are virulence factors that can suppress the host cell’s immune mechanisms [10]. Rice blast fungus invades adjacent cells through plasmodesmata, interferes with the host’s nutrient absorption, and leads to cell death—which, in turn, damages the tissue to form lesions. These lesions can produce conidia again and spread through the medium to initiate the next round of infection (Figure 1) [11].

### 2.2. Mechanisms of Action of PTI and ETI

**Figure 2:**
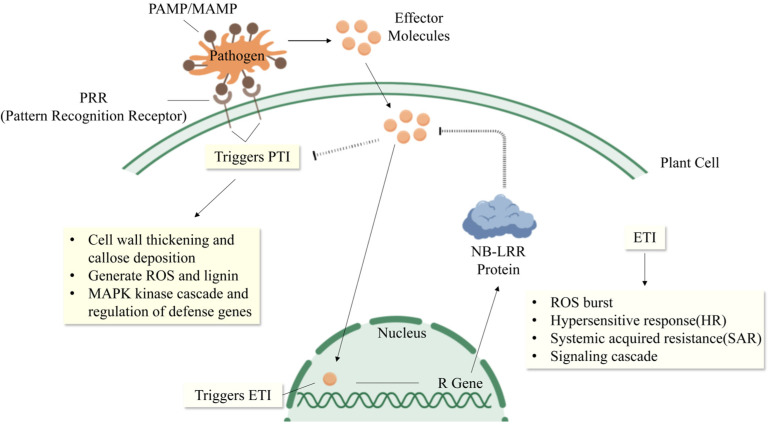
The reaction mechanism of PTI and ETI in rice’s innate immune system during rice blast infection [21]: PAMPs secreted by pathogenic bacteria are recognized by PRRs and trigger non-specific immune responses, resulting in a series of physiological responses, such as cell wall thickening and callose deposition, ROS and lignin production, MAPK cascade, and defense gene regulation, triggering the PTI defense response. The effector factors secreted by pathogenic bacteria specifically bind to the *R* gene and induce signal transduction, such as ROS burst, hypersensitive response (HR), systemic acquired resistance (SAR), and signal cascade, triggering the ETI defense response, and making effector proteins non-toxic.

With the evolution of pathogen populations, rice has gradually formed a variety of defense mechanisms in the host–pathogen co-evolution process. Two kinds of immune systems have been deeply studied and clarified [12]. One is the PTI induced by pathogenic molecules. PRRs on the surface of plant cells can recognize pathogen-associated molecular patterns (PAMPs) secreted by pathogens and trigger non-specific immune responses [13], including the production of reactive oxygen species (ROS) and lignin, as well as the regulation of mitogen-activated protein kinase (MAPK) cascades and defense genes, laying the foundation for the initiation of ETI while inhibiting the virulence of pathogens [14]. The second is the ETI induced by pathogenic bacteria effector proteins. In order to break through PTI, pathogenic bacteria have evolved effectors that can inhibit it, and plants have accordingly evolved *R* genes against effectors, triggering a highly specific immune response called ETI [15]. Rice blast infects rice through its own effectors. The rice *R* gene encodes a resistance protein (R-protein) that specifically recognizes the effector encoded by the rice blast avirulence gene (AVR) and induces signal transduction (such as MAPK cascades or Ca^+^ bursts) to trigger the defense response of ETI, and the effector becomes a ‘non-toxic factor’ (Avr protein), resulting in infection failure [16,17]. With the continuous evolution of pathogenic bacteria, the newly generated effectors are often unable to be recognized by the existing *R* genes. In order to cope with this challenge, rice will correspondingly evolve new *R* genes to identify these new effectors and trigger the second or even multiple effector-triggered immunity (ETI) responses, eventually forming a highly dynamic and complex plant immune network (Figure 2) [18].

So far, at least 146 rice blast *R* genes have been identified, and the NLR protein accounts for 86.4% of the total *R* genes in the cloned *R* gene pool, including *Pik* and its alleles *Pik-m*, *Pik-p*, *Piks*, *Pikh*, *Pike*, *Pi1*, *Pikg*, and *Pik-W25* in the *Pik* locus, *Pii* in the *Pi5* and *Pi5* loci, and *Pia* and *Pi-CO39* in the *Pia* locus [19]. Zhai et al. found that rice deubiquitinase *PICI1* is a key hub for plant immunity (PTI and ETI), which activates immunity by deubiquitinating and stabilizing methionine synthase and promoting the methionine–ethylene biosynthesis pathway. Plant immune receptor nucleotide-binding site (NBS)–leucine-rich repeat (LRR) proteins (such as *PigmR*) maintain the activity of methionine–ethylene immune cascade by protecting *PICI1* from the degradation of rice blast effector proteins [20].

## 3. Research Progress on Rice Blast Resistance Genes

In recent years, with the development of map-based cloning and genome-wide association analysis techniques, a large number of rice blast resistance genes and their related quantitative trait loci (QTLs) have been successfully excavated and identified. To date, more than 500 rice blast resistance QTLs have been identified, and more than 146 *R* genes have been identified, of which 38 have been molecularly characterized [19,22]. According to the mechanism of rice blast resistance, rice blast resistance-related genes can be divided into major resistance *R* genes, protein kinases, ubiquitin ligases, transcription factors, hormones, miRNA, and secondary metabolic enzymes.

### 3.1. Mechanism of Rice Blast Resistance Controlled by Major Resistance Genes (R Genes)

By molecular cloning of *R* genes and resistance QTLs, 38 major *R* genes distributed on 10 chromosomes (excluding chromosomes 5 and 7) were identified (Table A1) [23,24,25,26]. Based on the structural characteristics of the cloned primitive cell *R* gene and the predicted protein encoded by the resistance QTL, the encoded R proteins can be divided into the following types: The first is the nucleotide-binding site–leucine-rich repeat protein (NBS-LRR), for example, *pi9* is the first cloned major disease resistance gene and broad-spectrum disease resistance gene [27,28]. Zhou et al. identified 13 novel *Pi9* alleles with insertions or deletions from 361 blast-resistant rice varieties; among them, *Pi9-Type3*, *Type4*, *Type5*, *Type6*, *Type9*, *Type10*, and *Type11* can confer broad-spectrum resistance to rice blast [29]. Yu et al. identified a rice blast resistance gene *Pb2* encoding NLR protein by genome-wide association mapping [25]. *Piks* is a new NBS-LRR-*type R gene* cloned from the pick site [26]. The second type is receptor-like kinases (RLKs), such as *pid2* and *pi68*, which encode transmembrane receptor protein kinases with a serine/threonine kinase (STK) domain and have gene-to-gene resistance to rice blast race ZB15 [30,31]. *Pi65* is an LRR-RLK-type gene cloned from the resistant japonica rice variety Gang Yu 129; it is located on chromosome 11 near the telomere side of the *pik* locus [24]. The third class is the repeat-rich protein armadillo (ARM). *Ptr* is an atypical broad-spectrum disease resistance gene cloned in the resistant material Katy, and it is an allele of *pi-ta2*; its resistance allele *ptr-BHA* is effective against a variety of rice blast fungus races, such as the virulent races *IB-33* and *IE-1* [32,33]. The fourth category is proline-rich proteins. The first cloned *R* gene *pi21* encodes a proline-rich protein, which contains a putative heavy-metal-binding domain and several protein–protein interaction motifs. The deletion of the proline-rich motif in the dominant allele *Pi21* confers resistance to it, indicating that there is a unique mechanism of loss of function in partial resistance of plants [34]. Meanwhile, bsr-d1 encodes a *C_2_H_2_-type* transcription factor. Inhibition of *bsr-d1* expression can reduce the degradation of H_2_O_2_ and increase the accumulation of H_2_O_2_ in cells to enhance rice blast resistance [35].

**Figure 3:**
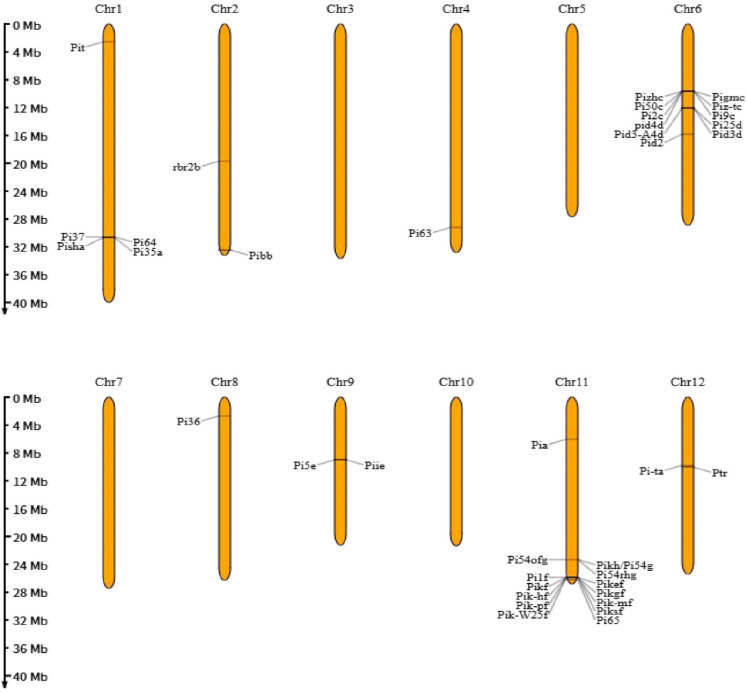
Distribution of the cloned R gene in chromosomes.

Most of the cloned resistance genes were clustered on chromosomes 1, 6, 9, and 11 (Figure 3), and most of them were obtained by map-based cloning of the known *R* gene alleles, while the resistance genes on other chromosomes usually existed in the form of independent genes [26,36,37,38]. In the *pi5*, *pik*, and *pia* gene clusters, all of the resistance genes are controlled by double-gene multi-alleles. One gene is responsible for pathogen recognition, which, in turn, activates the other gene to perform a disease resistance function [26,36,37,38]. Among the cloned resistance genes, the resistance gene loci determined by the LRR allele are mainly *pish*, *pi9*, *pid3*, *pi36*, and *pi-ta*. Some rice blast resistance genes, such as *pi2*, *pi5*, *pi9*, *pi54*, *pigm*, *pizt*, *pi50*, *pi56*, *pi64*, *pi68*, and *ptr*, showed broad-spectrum resistance to physiological races from various rice regions in the world, but most of these genes were only resistant to leaf blast. Only a limited number of *R* genes, such as *pb1*, *pi25*, *pi64*, and *pi68*, could effectively control panicle blast, among which *pi25*, *pi64*, and *pi68* were resistant to both leaf blast and panicle blast [29,31,39].

### 3.2. Resistance Mechanism of Rice Blast Controlled by Protein Kinases

As the first barrier against a variety of pathogens, the cell membrane has evolved a class of transmembrane receptors that can specifically recognize microbial-related molecules such as bacterial flagellin and fungal chitin [40]. A series of downstream immune responses are activated by receptor recognition, which is commonly referred to as PTI [13]. PTI plays a defensive role through a variety of immune pathways, including calcium influx, MAPK cascade reaction, RLK signal transduction, ROS burst, plant hormone signaling pathways, transcription reprogramming, cell wall enhancement, and synthesis and secretion of antibacterial compounds. Among them, protein kinases such as RLKs, receptor-like cytoplasmic kinases (RLCKs), MAPKs, and calcium-dependent protein kinases (CDPKs) play a central role in mediating rice blast resistance [41].

**Figure 4:**
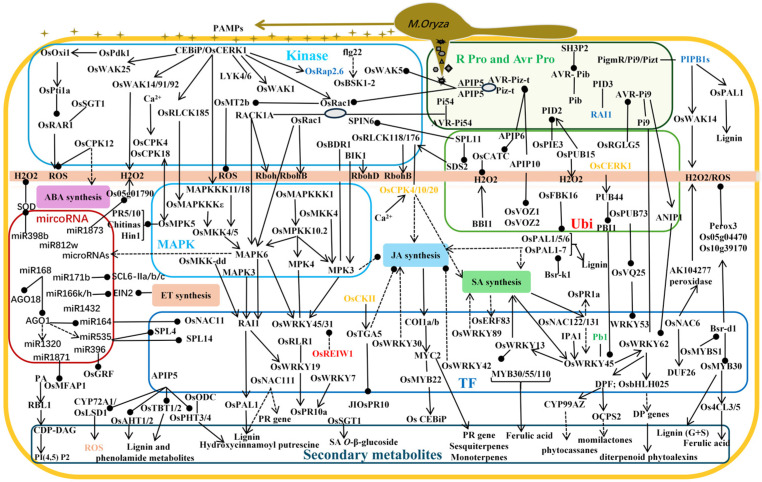
Mechanism of rice blast resistance mediated by disease-related genes: rice blast resistance working model mediated by protein kinase; ubiquitin ligase *APIP5-* and *APIP10*-mediated rice blast resistance working model; transcription factor *OsMYB30*-mediated rice blast resistance working model; rice blast resistance working model mediated by small RNAs miR1871 and miR369; plant hormone-mediated rice blast resistance working model. Note: triangular arrows represent promotion; the circular arrow represents inhibition; the straight line represents the interaction; the dotted line representation mechanism is not clear; the hollow circle represents several genes that can interact with one another.

RLKs transmit signaling molecules to cellular mechanisms and initiate signaling pathways. Genome-wide association analysis showed that there were 125 wall-associated kinase (WAK/WAKL) family members in the rice genome, of which 67 encoded WAK-type receptor kinases (WAK-RLKs), some of which were confirmed to be involved in rice blast resistance [42]. For example (Figure 4), *OsWAK1* can phosphorylate itself and the transcription regulator *OsRFP1*, and its expression can be induced by infection, mechanical damage, salicylic acid (SA), and methyl jasmonate (MeJA) treatment. The overexpression of *OsWAK1* significantly increased the resistance of rice to rice blast [43]. In addition, the early transcriptional regulation of *OsWAK91* and *OsWAK92* genes was induced by chitin under the regulation of the CEBiP receptor. *OsWAK91* is involved in the production of H_2_O_2_ and promotes the expression of rice blast resistance genes [44]. Overexpression of *OsWAK5* increased lignin content, enhanced rice blast resistance, and reduced the lesion area and pathogen load, and the defense-related gene oschitinase3 was significantly upregulated in overexpression lines [45]. For example, *OsWAK112d* negatively regulates rice’s resistance to rice blast infection and is a negative regulator of disease resistance [44]. *Pi21* encodes a protein rich in proline and cysteine [34], and *ptr* encodes a protein containing an armadillo repeat sequence, which is required for broad-spectrum blast resistance [33]. However, these two proteins have not been characterized in terms of their biochemical function. The *DR* genes/alleles that have been identified as having broad-spectrum resistance to rice blast include *bsr-k1* (broad-spectrum resistance-*Kitaake 1*), *bsr-d1* (broad-spectrum resistance-*Digu 1*), and *IPA1*. *Bsr-k1* encodes an RNA-binding protein that regulates RNA turnover of phenylalanine ammonia lyase (PAL) family genes [34]. *IPA1* encodes a squamous promoter-binding protein-like (SPL) transcription factor (TF), which activates defense-related *WRKY45* during rice blast fungus infection [46]. *OsBDR1* encodes a receptor-like kinase that phosphorylates the mitogen-activated protein kinase MPK3 to form a molecular module that negatively regulates the biosynthesis of jasmonic acid resistance hormones and terpene phytoalexins, thereby negatively regulating rice blast resistance [47].

U-box E3 ligase *SPL11* negatively regulates plant programmed cell death (PCD) and immunity. SPL11 cell death inhibitor *SDS2* encodes an S-domain receptor-like kinase. *SDS2* interacts with U-box E3 ligase *SPL11* and phosphorylates *SPL11*. *SDS2* mutation leads to reduced immune response and enhanced susceptibility to rice blast in rice. In addition, *SDS2* interacts with two receptor-like cytoplasmic kinases *OsRLCK118/176*, stimulating the production of reactive oxygen species by phosphorylating NADPH oxidase *OsRbohB*, thereby positively regulating immune responses and increasing resistance to rice blast. Therefore, the plasma membrane retention protein complex composed of *SDS2*, *SPL11*, and *OsRLCK118/176* controls the PCD and rice blast immune responses in rice [48].

CDPK is an important hub of plant calcium signal transduction and plays a crucial regulatory role in the immune response of rice. Studies [49] have shown that *OsCPK4* is involved in the signal cascade after pathogen recognition and exerts a defensive effect by inhibiting fungal penetration. When *OsCPK4* is constitutively activated, the defense signal can respond more quickly and strongly, significantly improving rice blast resistance. Another protein kinase, *OsCPK10* can not only autophosphorylate but also phosphorylate casein in a calcium-dependent manner, which may regulate disease resistance by activating SA and jasmonic acid (JA) dependent defense genes [50]. CDPKs regulate the downstream elements of the Ca signaling pathway. Studies on the overexpression and knockout of the rice CDPK gene *OsCPK12* showed that *OsCPK12* showed higher susceptibility to rice blast by reducing the accumulation of ROS and enhancing the sensitivity to abscisic acid (ABA) [51]. Li et al.’s study revealed the molecular mechanisms of two calmodulin kinases (*OsCPK18* and *OsCPK4*) in the IV subfamily of rice in regulating the balance of rice growth and disease resistance [52]. Studies [53] have shown that the calcium-dependent protein kinase *OsCPK18* is both a kinase and a substrate of the mitogen-activated protein kinase *OsMPK5*, and that the two can be phosphorylated. Transcriptome analysis showed that the *CPK18-MPK5* signaling pathway inhibited the expression of growth- and disease-resistance-related genes and negatively regulated rice blast resistance. Using Clustered Regularly Interspaced Short Palindromic Repeats (CRISPR/Cas9) to edit the phosphorylation sites of this process, rice lines with both high yield and disease resistance can be obtained [52]. The CRISPR/Cas9 system, as a powerful, efficient, and versatile genome-editing tool, has demonstrated great potential in breeding crops with enhanced disease resistance. By precisely targeting key susceptibility (S) genes or negative regulators, it enables the development of transgene-free, disease-resistant varieties, thereby helping to circumvent the regulatory challenges associated with genetically modified crops [54]. The Ca^2+^ sensor protein encoded by the rice susceptibility factor rod1 is endowed with rice blast resistance by the natural variant of rod1 caused by single-nucleotide deletion [55]. The expression level of the peroxidase-related gene *Os10g39170* plays a key role in rice blast resistance. Studies have shown that the expression of this gene can negatively regulate the level of H_2_O_2_ in rice, thereby weakening the resistance of rice to rice blast [35].

MAPKs are a class of intracellular serine/threonine protein kinases that are highly conserved in eukaryotic cells. Rice MAPK cascades play a crucial role in regulating rice’s resistance to rice blast. *OsMAPKKKε* is an early kinase in MAPK cascade, which interacts with and phosphorylates *OsMKK4*. *OsRLCK1* enhances the phosphorylation of *OsMAPKKKε* by phosphorylating and activating *OsRLCK185* (Figure 4) [56]. The expression of the *SMG1*-encoded kinase *OsMKK4* leads to the shift in metabolic flux from glycolysis to the biosynthesis of secondary metabolites, and it depends on *OsMPK6* to induce cell death, diterpene phytoalexin and lignin synthesis, etc., to enhance rice blast resistance, but it does not cause extracellular ROS production [57].

Some other types of kinases also play an important role in the process of rice blast resistance. For example, casein kinase II *OsCk2α2* can interact with the negative regulator of rice blast resistance *OsTGA5*, reduce the transcriptional inhibition of *OsTGA5*, and enhance the resistance to rice blast [58]. The interaction or antagonism between *RAR1* and *SGT1* plays an important role in the immune system of plants [57].

### 3.3. Partial Resistance Mechanism of Rice Blast Controlled by Ubiquitin Ligases

The ubiquitin–proteasome system (UPS) determines the stability of intracellular proteins and plays an important role in the PTI and ETI immune responses against rice blast [59]. *APIP6*, *APIP10*, *OsRGLG5*, and *OsBBI1* of the RING family belong to E3 ubiquitin ligase (Figure 4). *APIP6* promotes flg22-induced ROS accumulation by targeting the rice blast fungus effector *Avr*-*piz t* and rice’s own *OsCatC*. In vitro, *APIP6* ubiquitinates *Avrpiz-t* in the form of a homodimer, and *Avrpiz-t* feedback can inhibit the enzyme activity of *APIP6* [60,61]. *APIP10* can negatively regulate the basic resistance to rice blast fungus and positively regulate the ETI response mediated by the NLR protein *piz-t* by ubiquitinating the transcription factors *OsVOZ1* and *OsVOZ2* [62]. *OsRGLG5* degrades the effector *Avrpi9* via ubiquitination during rice blast fungus infection. *Avrpi9* can also initiate feedback to reduce the stability of the *OsRGLG5* protein [63]. By promoting the deposition of H_2_O_2_ and phenolic compounds in the cell wall, OsBBI1 significantly thickens the cell wall, thereby conferring broad-spectrum resistance to rice blast [64].

The UPS plays an important role in regulating plants’ PTI and ETI immune responses by maintaining the stability of immune-related proteins in the regulation of rice blast resistance. Through the identification of rice blast-sensitive genes, it was found that the F-box E3 ligase *OsFBK16* was the core interaction protein of the phenylalanine ammonia lyase family *OsPAL1~7*, revealing the molecular mechanism of *OsFBK16* negatively regulating rice blast resistance by degrading *OsPALS* [65]. The E3 ubiquitin ligase *OsPIE3* negatively regulates rice blast resistance mediated by the receptor-like kinase *pid2*. *OsPIE* promotes the recruitment of *pid2* from the plasma membrane to the nucleus by changing the subcellular localization of *pid2*, and it relies on the ubiquitin–protease system to achieve the degradation of *pid2*. Compared with the wild type, the resistance of the *OsPIE3* deletion mutant to the rice blast fungus race *ZB15* is enhanced, while the resistance of the *OsPIE3* overexpression line is significantly weakened [66].

### 3.4. Mechanism of Rice Blast Resistance Controlled by Transcription Factors

TF, also known as trans-acting factor, is activated by intracellular signal transduction when rice is infected with rice blast fungus, and then it regulates the expression of a series of stress-response genes, thereby mediating the resistance to rice blast [42]. At present, it has been reported that there are three main types of transcription factors related to rice blast resistance: WRKY transcription factors, MYB transcription factors, and NAC transcription factors (Figure 4).

WRKY transcription factors can directly or indirectly participate in two or more stress responses and have important biological functions. The transcription factor *OsWRKY31* is regulated by phosphorylation and ubiquitination; it can form a ternary complex with *OsMKK10-2* and *OsMPK3* and interact with them for phosphorylation, which can increase the expression of disease resistance-related genes and the accumulation of SA and JA; it can also be ubiquitinated by the E3 ubiquitin ligase *OsREIW1* to promote its degradation, but the phosphorylation of *OsWRKY31* alleviates its degradation [67].

*WRKY45* is a key regulator involved in the immune response to rice blast and bacterial blight in rice. In the absence of pathogen infection, the ubiquitin ligase PUB44 is phosphorylated in an oscerk1-dependent manner, which, in turn, mediates the degradation of *PBI1*. The phosphorylation of *WRKY45* by MAPK and the degradation of *PBI1* completely activate *WRKY45* [68]. *OsWRKY45-1* and *OsWRKY45-2* are two alleles, and their coding products are only 10 amino acids different. Both alleles play a positive regulatory role in rice blast resistance [69]. *OsWRKY45-1* enhances rice’s resistance to pathogens by regulating the expression of defense-related genes and promoting the accumulation of SA and JA. *WRKY45-2* can transcriptionally activate *WRKY13*, while *WRKY13* directly inhibits *WRKY42*, which constitutes a sequential transcriptional regulatory cascade. Finally, *WRKY42* negatively regulates the defense response of rice to rice blast fungus by inhibiting genes related to the JA signaling pathway (Figure 4) [68].

Studies have shown that *bsr-d1* can indirectly negatively regulate the expression of *OsMYB30*, and that *OsMYB30* promotes the significant thickening of epidermal sclerenchyma cells by regulating the accumulation of 4-coumaric acid, thereby inhibiting the invasion of rice blast fungus at the early stage of infection [70,71].

The *bZIP* transcription factor *APIP5* is a negative regulator of cell death and rice immunity. It was found that, on the one hand, *APIP5* can form homodimers and interact with the fungal effector *Avrpiz-t* in the cytoplasm to promote the entry of rice blast fungus into the dead-body nutrition stage. The resistance gene *Piz-t* promotes the accumulation of APIP protein by binding to *APIP5*, thereby inhibiting cell necrosis and blocking the transformation of rice blast fungus from the living nutrition stage to the dead nutrition stage. On the other hand, *APIP5*, as a transcription factor, can directly bind to the promoters of cell wall-related kinase *OsWAK5*, cytochrome P450 CYP72A1, putrescine hydroxycinnamoyl transferase *OsPHT4*, agmatine hydroxycinnamoyl transferase genes *OsAHT1/OsAHT2*, tryptamine hydroxycinnamoyl transferase genes *OsTBT1/OsTBT2*, and tyramine hydroxycinnamoyl transferase genes *OsTHT1/OsTHT2*, inhibiting their transcription [45].

NAC transcription factors are among the plant-specific transcription factors. *OsNAC6* is one of them, which is highly similar to the ATAF subfamily; it is induced under abiotic stress and fungal disease infection, and its overexpression can significantly reduce the degree of rice blast infection [72]. Sun et al. identified two homologous genes of rice NAC transcription factors, *OsNAC122* and *OsNAC131*, which can regulate the expression of other defense and signal-related genes and play an important role in rice’s disease resistance [73]. The rice transcription inhibitor *MYBS1* binds to the *bsr-d1* promoter and reduces the expression of *bsr-d1*, which inhibits the degradation of hydrogen peroxide, leads to the accumulation of H_2_O_2_, and confers resistance to rice blast [4]. *ERF922* encodes an Apetala2/ethylene response factor (AP2/ERF) transcription factor and is induced by rice blast. Knockout of *ERF922* can enhance the resistance of rice to rice blast, indicating that this gene plays a negative regulatory role in disease resistance [74].

### 3.5. Partial Resistance Mechanism of Rice Blast Controlled by microRNA

MicroRNAs (miRNAs) can bind to Argonautes (AGO protein family) to form the RNA-induced silencing complex (RISC), which targets specific mRNAs in a sequence-complementary manner and inhibits gene expression via transcriptional cleavage or translational inhibition [75]. Studies have shown that miRNA is one of the important mechanisms regulating plants’ growth, development, and defense through target gene expression [76,77]. For example, the polycistronic miRNAs of the miR166 family members mir166k and miR166h play the role of positive rice immune regulators by regulating the expression of the ethylene signaling pathway gene EIN2 [78]. Recently, Li et al. found that overexpression of mir171b in rice plants (Ox171b) not only enhanced their rice blast resistance and defense response but also delayed their heading date. The mutants of the target genes *SCL6-IIa*, *SCL6-IIb*, and *SCL6-IIc* of *miR171b* have similar phenotypes to *Ox171b*, indicating that mir171b coordinates yield, growth period, and rice blast resistance by regulating the *SCL6-II* gene (Figure 4) [79].

Mir168 targets AGO1, a key component of the RISC. Overexpression of mir168 in rice significantly inhibited the mimic target of AGO1-miR168 (mim168), weakened the negative regulation of miR168 on AGO1, and thereby enhanced the resistance of rice to rice blast, while promoting the increase in tillers, shortening the growth period, and increasing the yield [80].

Not only have recent studies have found that mir168 targets AGO1, an important component of the RNA-induced silencing complex, but also bioinformatics prediction and gene expression analysis have confirmed that *OsAGO18* is a potential target gene. KEGG enrichment analysis showed that osmir168 was involved in many key biological processes, such as plant hormone signal transduction and plant–pathogen interaction [81]. *OsMFAP1* encodes a microfilament-related protein located near the cell wall and can positively regulate the PTI response. However, mir1871 reduced rice blast resistance by inhibiting the expression of *OsMFAP1*. Inhibition of mir1871 function and overexpression of OsMFAP1 can enhance the PTI response of rice, thereby significantly improving its resistance to rice blast [82]. Mir396 can target the expression of growth regulators and negatively regulate the resistance of rice to rice blast by inhibiting a variety of *OsGRF*, thereby differentially controlling the growth and yield of rice [83].

### 3.6. Partial Resistance Mechanism of Rice Blast Controlled by Plant Hormones

Plant hormones are signaling molecules that are produced at extremely low concentrations in plants and can trigger physiological effects; they are involved in the regulation of various stages of plants’ growth and development. With the deepening of plant biology research, it has been confirmed that plant hormones play an important role in plants’ disease resistance and stress resistance [84].

Phospholipids are essential components of biofilm formation and are involved in various biological and physiological processes, including cell development and responses to biotic and abiotic stresses. It has been found that the cytidine diphosphate diacylglycerol synthase RBL1 is a key enzyme in phospholipid biosynthesis; as a cytidine diphosphate glycerol (CDP-DAG) synthase, it plays an important role in the synthesis of phosphatidylinositol and can regulate programmed cell death and immune response (Figure 4) [85]. In addition, phospholipid inositol diphosphate (PtdInsP_2_) is localized in rice as a disease susceptibility factor, especially in the enrichment of infection-specific structures, such as the biotype-interacting complex (BIC) and extra-invasive hyphal membrane (EIHM) [85]. Zhou et al. found that *bsr-k1* encodes a tetratricopeptide repeat (TPR) protein with RNA-binding activity, which can bind to the mRNAs of multiple members of the immune response-related *OsPAL* family (*OsPAL1-7*), promote the folding and degradation of these mRNAs in rice, and weaken the immune response of rice by reducing the synthesis of lignin. Single-base mutation leads to the loss of function of the *bsr-k1* protein, which greatly reduces its ability to bind to *OsPAL* gene mRNA, thereby significantly increasing the mRNA expression of *OsPAL* family genes, promoting lignin synthesis, and enhancing the immune response. This variation confers broad-spectrum resistance to rice [34]. Meng et al. [86] identified an activator *OsbHLH6* that activates JA signaling and inhibits SA signaling pathways within 24 h after rice blast infection, thereby reducing rice blast resistance. After 24 h, osnpr1 induced *OsbHLH6* export from the nucleus to the cytosol and inhibited *OsbHLH6*-mediated JA and SA signaling activation, thereby conferring resistance to rice blast. *OsTrxm* plays an important role in the redox regulation of chloroplasts, and RNAi treatment will increase the content of H_2_O_2_. The interaction between *OsMESL* and *OsTrxm* can synergistically negatively regulate the production of ROS and JA [87].

JA plays an important role in plants’ growth, development, senescence, and various stresses. JA participates in and regulates the disease resistance mechanisms of plants in the form of defense signaling molecules. For example, when rice blast fungus infection occurs, overexpression of the transcription factor *WRKY30* activates the expression of the JA synthesis-related genes *LOX* and *AOS2*, the pathogen-related genes *PR3* and *PR10*, and the accumulation of endogenous JA under the stress of pathogenic fungi [88].

At 22 °C, JA synthesis decreased, while at 28 °C, rice blast fungus induced JA synthesis and activated the JA signaling pathway, which improved rice disease resistance [71]. Therefore, the application of MeJA in a warm environment can improve the resistance of rice to rice blast by activating the disease resistance mechanism, which makes it a commonly used drug for the prevention and control of rice blast.

ABA plays an important role in many physiological processes of plants’ life cycle. ABA is closely related to the establishment of seed dormancy, seed germination, root development, reproductive growth, abiotic stress response, resistance to pathogen invasion, and induction of stomatal closure [89]. ABA is considered to be a negative regulator of rice blast resistance. Exogenous application of ABA and low temperatures will increase the sensitivity of rice blast-susceptible varieties. So far, the molecular and biochemical mechanisms associated with ABA-mediated susceptibility to rice blast remain unclear [90]. Due to the inhibition of systemic acquired resistance (SAR) mediated by the SA, JA, and ET signaling pathways, excessive production of ABA in plants may adversely affect their disease resistance [91]. For example, the ETI signaling pathway regulator *OsEIL1* in rice can bind to the promoters of the nicotinamide adenine dinucleotide phosphate oxidase gene *OsRboh* and the JA biosynthesis gene *OsOPR4* and activate their expression, thereby promoting ROS accumulation [92].

### 3.7. Resistance Mechanism of Rice Blast Controlled by Secondary Metabolites

Secondary metabolites play a crucial role in plants’ resistance to external invasion; they are not only an important guarantee for plant life activities—such as plant hormones (IAA, GA), photosynthetic pigments (carotenoids), and anthocyanins (volatile substances attract insects to pollinate)—but also a protective barrier for plants against external threats, such as polyphenol tannins, which are toxic to animals or microorganisms but harmless to plants themselves. Secondary metabolites often become important drugs or industrial raw materials. For example, secondary metabolites (flavonoids) regulate oxidative stress. Flavonoids can balance ROS and directly kill pathogens, while avoiding the impact of excess ROS on host cells. Superoxide dismutase (SOD) is involved in the synthesis of lignin and the crosslinking of cell wall components to enhance the structural resistance of plants’ physical barriers. SOD, aldehyde oxidase (AOX), catalase (CAT), etc., can remove excessive ROS and maintain intracellular homeostasis [87].

PAL is a key rate-limiting enzyme in phenylpropanoid metabolism; it regulates the rate of phenylalanine entering the phenylpropanoid metabolic pathway and affects the accumulation of secondary metabolites such as lignin, flavonoids, isoflavones, alkaloids, and benzoate glycosides in plants. The expression of the agmatine hydroxycinnamoyl transferase genes *OsAHT1*/*OsAHT2* and tryptamine hydroxycinnamoyl transferase genes *OsTBT1*/*OsTBT2* was induced by rice blast infection; the contents of phenolic metabolites and lignin in rice were increased, the fungal biomass was decreased, the transcription levels of the defense-related genes *OsChitinase3* and *OsPR10* were increased, and the resistance of rice to rice blast was enhanced [93]. As a damage-associated molecular pattern (DAMP), the receptor *OsCERK1* on rice cell membranes can recognize the endoglucanase secreted by rice blast fungus to degrade the hemicellulose of rice cell walls and activate rice’s cellular immune response [94]. Shen et al. used a metabolite-based genome-wide association study (GWAS) to find that the tyramine hydroxycinnamoyl transferase genes *OsTHT1* and *OsTHT2* are involved in the metabolic accumulation of hydroxycinnamic acid, hydroxycinnamaldehyde, hydroxycinnamyl alcohol, flavonoids, and phytoalexin, thereby mediating disease resistance. This is related to the enhancement of phytoalexin and the metabolic reprogramming of the phenylpropanoid pathway. In addition, the expression of the defense signal-related genes *OsPR1a*, *OsPR5*, *OsEDS1*, and *OsCht1* increased with the increase in H_2_O_2_ content in overexpression lines, while the expression of defense signal-related genes decreased with the increase in H_2_O_2_ content in double-knockout lines [95].

## 4. Rice Blast Resistance Breeding

In the 1980s, rice varieties planted in China carried rice blast resistance genes, which could effectively prevent and control rice blast fungus at that time. However, with the emergence of new pathogenic strains (Table A2 and Table A3), these genes gradually lost their effectiveness, resulting in a decline in rice’s disease resistance. In view of the rapid variation in rice blast fungus, scholars at home and abroad have carried out a lot of research to explore how to effectively use resistance genes to maintain persistent and stable resistance [96]. Researchers have cloned a number of broad-spectrum disease-resistant *R* genes and conducted in-depth research in combination with high-quality varieties [97]. At present, the breeding of resistant varieties can be classified into conventional breeding, molecular-assisted selection breeding, genome-wide selection breeding, and gene-editing breeding.

### 4.1. Induce Blast Resistance Through Marker-Assisted Selection Breeding

Rice blast resistance breeding mainly adopts cross and backcross methods, including conventional breeding and molecular breeding. Among them, conventional breeding introduces disease resistance genes into varieties through hybridization, such as Huanghuazhan [96], but it has the disadvantages of a long cycle and heavy workload. In recent years, with the development of biotechnology, more and more genes related to rice blast resistance have been cloned. The application of new technologies such as gene editing has brought a breakthrough opportunity for disease resistance breeding. For example, the hybrid rice Tianyou 998 not only has strong resistance to rice blast and bacterial blight but also has excellent rice quality [98]. Kuang Haochi et al. [99] developed an indica restorer line Luhui 37 with high yield and strong resistance through conventional breeding, and they further developed Chuanyouxiang 37. Despite the limitations of conventional and hybrid breeding techniques, they are still the main means of rice blast resistance breeding in China [100].

### 4.2. Induce Blast Resistance Through Marker-Assisted Selection Breeding

Marker-assisted selection (MAS) technology can transfer one or more target genes to high-quality varieties in a more lasting combination, thus accelerating the integration of rice blast *R* genes in traditional breeding [101]. By identifying and combining multiple resistance genes, MAS has developed rice lines with broad-spectrum and durable resistance [102]. Transgenic technology introduces known resistance genes into excellent rice varieties through particle bombardment or Agrobacterium-mediated methods to rapidly improve their resistance to rice blast [19].

Jia et al. [101] have made significant progress in identifying the markers of the Rita gene. In order to locate the *Pi66 (t)* gene on chromosome 11, the new PCR markers *WRKY41*, NBS-LRR-970-1, and NBS-LRR-970-2 were developed, and their separation effect with *pi66 (t)* was verified, providing an important tool for related gene screening. The functional marker GM was used to hybridize *R6888* carrying *pigm* and Luoyang 69 carrying *Bph6*/*Bph9*. Combined with molecular genotype screening and agronomic trait phenotypic evaluation, the F pedigree with stable inheritance and excellent traits was screened. Subsequently, the excellent lines were hybridized with thermosensitive genic male sterile (TGMS) lines, the gene pyramiding of *Bph6*, *Bph9*, and *pigm* was successfully achieved, and the restorer lines with high resistance to both rice blast and brown planthopper were developed [103].

**Figure 5:**
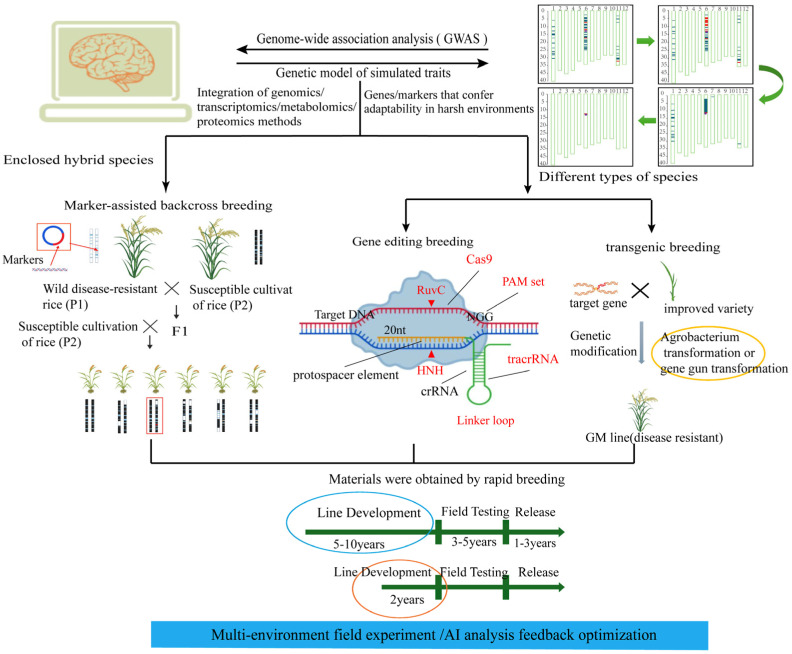
High-quality target genes were selected by rice breeding technology [131,132]. According to the goals and needs, artificial intelligence-assisted breeding and genomics-assisted breeding were used to predict excellent combinations and excellent gene mapping, and then multiple *R* genes were combined by molecular marker-assisted breeding and gene pyramids, and markers were used to quickly screen and locate *R* genes. Then, through gene editing and transgenic breeding, gene modification was carried out to give rice a new shape. Finally, the target new varieties were screened by multi-environment field testing.

In addition, MAS technology is widely used in the management of bacterial blight (BB) in India. Through MAS technology, three BB resistance genes (*Xa21*, *Xa13*, and *Xa5*) were introduced into aromatic short-grain rice (HUR917), and rice varieties with broad-spectrum resistance to BB were successfully cultivated, which further proved the utility of MAS in accelerating trait infiltration [104] (Figure 5).

Ye et al. [105] introduced the rice blast resistance genes *pigm* and *pi-ta*, as well as the aroma gene *Badh2*, into high-quality HR1212 japonica rice via a molecular marker-assisted selection backcross method. The obtained introgression lines not only significantly enhanced the resistance to panicle blast but also improved the yield, while maintaining the excellent taste quality similar to HR1212. The new TPAP functional marker developed by Mao et al. [106] achieved the efficient and accurate detection of these resistance genes and significantly shortened the breeding cycle. Using MAS, three broad-spectrum blast resistance genes (*Pita*, *PibI*, and *Pid2*) were simultaneously introduced into the high-yield japonica rice variety YF47, and an excellent new line (YJ144) was bred. The leaf blast index and the incidence of panicle blast were significantly reduced, and the yield and appearance quality were improved. Wang et al. [107] successfully introduced the broad-spectrum rice blast resistance gene *Pigm* into the excellent thermosensitive genic male sterile line LK638S through molecular marker-assisted selection, which significantly improved its disease resistance, and cultivated an improved line LZ36S with high resistance and excellent agronomic traits and yield potential, along with its derivative hybrid varieties. At the same time, the effectiveness of molecular marker-assisted selection in overcoming the drag of gene linkage and achieving the accurate introduction of target genes was verified, providing an efficient strategy for rice disease resistance breeding. In addition, qi et al. [108] successfully mapped and cloned the new rice blast resistance gene *Pik-W25* by bulked segregant analysis (bsa-seq); its resistance is mediated by *Pik-W25-1* and *Pik-W25-2*, which can recognize *AvrPik-C* and fill the gap in the existing *Pik* allele recognition spectrum. The results of the field experiments showed that the introduction of the *Pik-W25* allele enhanced disease resistance without sacrificing agronomic traits.

These studies emphasize that marker-assisted breeding methods provide new ideas for rice blast resistance breeding in strengthening rice blast management and rice breeding, with the potential to greatly improve resistance to multiple diseases.

### 4.3. Inducing Rice Blast Resistance by Genomics-Assisted Breeding

Genomics-assisted breeding (GAB) encompasses genome-wide selection breeding and molecular marker-assisted breeding. Genomic selection (GS) breeding is the use of whole-genome sequencing to predict quantitative traits that depend on genotype, environment, and control by minor genes [109]. Compared with molecular-assisted markers, genome-wide selective breeding has higher efficiency in quantitative traits regulated by multiple genes, and it is not limited by conventional species or hybrids in practical applications. Since the first reference genome of Nippon bare was published in 2005, the Rice Atlas database has been constructed by deep genome resequencing of 6044 modern rice varieties [110,111].

Through genome resequencing of 200 japonica rice varieties in Zhejiang and other places, *piz-t* was identified by GWAS and XPCLR as the main rice blast resistance gene of japonica rice varieties in Central China and had excellent effects on yield and rice quality, leading to the cultivation of varieties *XY99* and *JXY1* with excellent taste, high yield, and broad-spectrum resistance to rice blast [112].

The results showed that 546 rice germplasm resources in Northeast China were analyzed by resequencing, and the genome-wide association analysis of 22 agronomic traits was carried out to construct the molecular model of Northeast China. On this basis, a series of molecular modules—such as the heading date gene Hd1; grain type genes *GW5*, *GS3*, and *GL7*; rice blast resistance genes *pita*, *ptr*, and *pib*; yield trait loci *qSB2*, *qSB8*, and *qSB10*; and lodging resistance gene *SCM2*—were concentrated in the same variety or breeding material, and the rice variety ‘Zhongkefa No.5’ was successfully bred, achieving the synergistic improvement of high yield, high quality, disease resistance, and lodging, marking breakthrough progress in multi-trait molecular modules [113]. *Pi1*, *pi2*, *piz*, *pi9*, and *pigm* were introduced into the genome of *Kongyu131* by continuous backcrossing and genome-wide selection to produce multi-line varieties with blast resistance [114]. The whole-genome molecular marker-assisted selection technique combined with the conventional backcross breeding method was used to improve the rice blast resistance of Chaoyouqian R900, and an improved R900 line with homozygous resistance genes was obtained. This variety retained its original potential and agronomic traits, and its rice blast resistance was also improved [115].

Feng et al. [116] successfully identified and deeply analyzed the rice blast resistance gene *SBRR1* and its elite allele *SBRR1-R* through a GWAS, revealing its great potential in rice disease resistance breeding. *SBRR1-R* can significantly upregulate gene expression through the specific binding of its promoter to the transcription factor bHLH57, thereby enhancing resistance to rice blast fungus.

Julian R Greenwood et al. [117] successfully identified and verified the novel functional alleles of the rice blast resistance genes *Ptr* and *Pia* via GWAS allele analysis of 3000 rice genomes. The newly identified *Ptrb* allele confers specific resistance to the specific rice blast strains Mo15-23 and Mo15-24. In addition, two new functional alleles of the Pia gene (*RGA4* and *RGA5*) were also identified; they had amino acid changes in the HMA effector-binding domain and could recognize *AVR1-CO39* and *AVR-Pia* effectors, but they did not show a differential resistance response to rice blast isolates.

In the process of molecular breeding, balancing rice yield, disease resistance, and quality is a huge challenge. Using genome-wide selection breeding to improve existing rice varieties rapidly improves their rice blast resistance, yield, and quality, and it can be directly applied to production, providing an important guarantee for food security.

### 4.4. Induce Blast Resistance Through Gene-Editing Breeding

The rise in gene-editing technology provides a new technical platform for broad-spectrum resistance breeding against rice blast. The CRISPR/Cas9 system, as a rapidly developing gene-editing tool, has shown broad application prospects in the breeding of rice and other crops [97].

The *bsr-d1* gene of Jigeng88 (JG88) was knocked out by CRISPR/Cas9 technology, and the mutant (KO) was obtained. After inoculation with *ZA41*, the level of ROS in the mutant increased significantly, indicating that the *bsr-d1* gene mutation could enhance the resistance to rice blast [118]. In another study, the *SD1* gene was edited by CRISPR/Cas9 and introduced into the variety ‘Shuihuai 119’ by Agrobacterium-mediated transformation to obtain homozygous mutants. Phenotypic analysis showed that the mutant not only had significantly improved agronomic traits but also had improved resistance to rice blast [119] (Figure 5).

The triple-knockout mutants of the susceptibility genes *pi21*, *bsr-d1*, and *xa5* were constructed by CRISPR/Cas9 technology in the background of japonica rice NPB. Studies have shown that the broad-spectrum resistance of rice to rice blast and bacterial blight after triple-gene mutation is significantly higher than that of single-gene mutation [120].

Against the background of the indica TGMS rice line *Longke638S* (LK638S), the susceptibility genes *pi21*, *bsr-d1*, and *OsERF922* were knocked out by CRISPR/Cas9, and the varieties with enhanced resistance to rice blast and no adverse effects on agronomic traits were successfully cultivated [121] (Figure 5). Some scholars have used CRISPR/Cas9 gene-editing technology to introduce the genes *EPSPS*, *OsLecRK1*, *Bph14*, *Cry1C*, *Xa23*, and *Pi9* into excellent rice varieties to enhance their resistance to rice blast [122].

Xu et al. [123] constructed a co-editing vector pC1300-2 × 35S: Cas9-g^Pita^-g^Pi21^-^gERF922^ via CRISPR/Cas9-mediated site-directed editing of the rice blast susceptibility genes *Pita*, *Pi21*, and *ERF922*, obtaining *Pi21* single-mutant homozygous lines and *Pita*, *Pi21*, and *ERF922* triple-mutant homozygous lines with the long-grain japonica restorer line L1014 as the receptor. The results showed that the rice blast resistance of homozygous mutants was improved. Li et al. [124] identified the U-box E3 ubiquitin ligase OsPUB41 as promoting the ubiquitination of OsPALs and further leading to the protein degradation of these key enzymes, which directly inhibited the accumulation of lignin. CRISPR/Cas9 was used to knock out the U-box E3 ubiquitin ligase *OsPUB41* that negatively regulates rice blast resistance, so that *OsPALs* proteins were stabilized and accumulated, thereby enhancing the biosynthesis of lignin and, ultimately, significantly improving the disease resistance of rice. Sha et al. [85] identified the key role of rice’s *RBL1* gene in balancing broad-spectrum disease resistance and yield. RBL1 was finely regulated by CRISPR/Cas9 technology, and an excellent allele *RBL1*^Δ12^ was obtained. This gene confers broad-spectrum resistance to rice blast fungus, bacterial blight fungus, and rice false smut fungus, which not only does not lead to a decrease in yield but also shows a significant yield advantage in high-incidence areas. Yu et al. [125] artificially explored the specific function of the *bZIP* transcription factor *OsbZIP76* and constructed *OsbZIP76*-knockout rice lines via CRISPR/Cas9. Compared with the wild type, *OsbZIP76* knockout resulted in a significant increase in the susceptibility of rice to bacterial blight and rice blast. It was revealed that *OsbZIP76* plays an important role in plants’ immune activation by positively regulating the expression of defense genes and mediating ABA sensitivity.

Cláudio Bezerra [126] successfully knocked out two specific susceptibility genes in rice using CRISPR/Cas9 gene-editing technology, developing a new rice line with significant resistance to rice blast. Compared with traditional breeding methods, gene-editing technology can introduce resistance traits more accurately and efficiently, and it can overcome the problem of easy loss of resistance in existing resistant varieties.

In summary, CRISPR/Cas9 technology for rice blast resistance breeding shows great potential in the development of new varieties with broad-spectrum disease resistance; it not only provides an important tool for molecular breeding of rice disease resistance but also opens up a new pathway for the enhancement of disease resistance in other crops.

### 4.5. Assist Rice Blast Resistance Breeding Through AI Technology

Artificial intelligence protein structure prediction tools (such as AlphaFold2, Rosetta Fold, and AlphaFold3) have important potential in the identification and sequencing of disease resistance genes [127]. Advances in genome sequencing and assembly technology have promoted the development of pangenomes, enabling them to be constructed in eukaryotes, fungi, plants, and animals [128]. Compared with a single genome, pangenomes can identify new genes, structural variations, and the presence/absence of variations, and they can track the origin and evolution of gene families related to important agronomic traits such as disease resistance [129].

The interaction between the rice blast *Avr* gene and rice *R* gene was predicted by AlphaFold3. Structural analysis showed that *Avr-pita* could bind to *Pi-ta* through the LRR domain, while *Avr-pik* recognized *OsHIPP19* using the heavy-metal-associated (HMA) domain and interacted with *pi9* through the adjacent LRR domain. Shang et al. [130] systematically analyzed the NBS-LRR genes in the rice pangenome and found that most of the NLR genes exist in their wild ancestor species. Although pangenomics has made great progress, it also faces great challenges in data analysis, computing resources, and experimental verification while revealing genomic diversity [128]. Therefore, these findings enhance the understanding of plant–pathogen interaction mechanisms and contribute greatly to rice blast resistance breeding programs [101].

## 5. Conclusions

In recent years, significant progress has been made in rice blast resistance mechanisms and breeding technology. The application of advanced techniques—such as the cloning of broad-spectrum disease resistance genes, the identification of susceptibility genes, and gene editing—has laid a solid foundation for the cultivation of disease-resistant rice varieties. The discovery of disease resistance genes and susceptibility genes not only improves the accuracy of breeding but also deepens the understanding of the immune mechanisms of rice [133]. The cultivation of disease-resistant rice varieties is a rapidly developing field, which benefits from the continuous advancement of genetics and molecular technology. The gene-pair gene model provides a theoretical basis for the interactions between plants and pathogens, and it highlights the direction for the development of breeding strategies to enhance the resistance of rice to major diseases [127]. However, the rapid variation in pathogenic bacteria, the incomplete analysis of some *R* gene resistance mechanisms, and the balance between disease resistance and agronomic traits are still the main challenges.

## 6. Future Prospects

Although significant progress has been made in the identification and application of *R* genes, the rapid evolution of rice blast fungus has led to the collapse of *R*-gene-mediated resistance. Future research should strengthen multi-trait collaborative genome selection and develop high-resolution, high-throughput functional markers to improve breeding efficiency and resistance persistence. Future research should focus on the systematic analysis of the interaction mechanisms between pathogens and hosts, especially via an in-depth study of the synergistic effects of PTI and ETI. At the same time, atomic-scale insights into the R-*Avr* recognition mechanism in rice and other crops have demonstrated the feasibility of designing synthetic *R* genes with extended pathogen recognition spectra [134]. Since breeding practices are usually centered on simultaneously improving yield and disease resistance, there may already be some crop varieties that have both of these advantages and contain similar genes. However, these varieties and the genes they carry have not been fully studied and elaborated [40]. In terms of opportunities, the integration of cutting-edge technologies such as gene editing, genomics-assisted breeding, rapid breeding, and artificial intelligence will provide unprecedented possibilities for the efficient and accurate breeding of new disease-resistant varieties. Although the CRISPR/Cas9 system still faces challenges such as off-target effects and commercialization barriers, advances in Cas9 protein engineering, optimized gRNA design, and the introduction of novel systems such as Cas13 are gradually addressing these limitations. In the future, by integrating functional genomics, synthetic biology, and systems biology, CRISPR/Cas9 is expected to enable the simultaneous improvement of multiple traits. The future directions of research should focus on the construction of a comprehensive breeding system with broad-spectrum, durable resistance and excellent agronomic traits so as to realize efficient transformation from laboratory results to large-scale promotion, thereby playing a key role in ensuring global rice production safety and food supply.

## Data Availability

All data are shown in the main manuscript.

## Abbreviations

The following abbreviations are used in this manuscript:

PTI	PAMP-triggered immunity
PAMP	pathogen-associated molecular pattern
ETI	elicit effector-triggered immunity
PRRs	Pattern recognition receptors
NLR	nucleotide-binding domain leucine-rich repeat
PAMP	Pathogen-associated molecular patterns
aROS	reactive oxygen species
MAPK	mitogen-activated protein kinase
AVR	avirulence gene
NBS	nucleotide binding sites
LRR	leucine rich repeat
QTL	quantitative trait locus
NBS-LRR	nucleotide binding site–leucine-rich repeat protein
RLKs	Receptor-like kinase
STK	serine/threonine kinase domain
ARM	Armadillo
RLCK	receptor-like cytoplasmic kinase
CDPKs	Calcium-Dependent Protein Kinases
WAK-RLKs	WAK-type receptor kinases
SA	salicylic acid
MeJA	Methyl Jasmonate
WAK/WAKL	Wall-associated kinases
PAL	Phenylalanine Ammonia-Lyase
SPL	Squamosa promoter-binding protein-like
TF	transcription factors
JA	jasmonic acid
ABA	Abscisic acid
UPS	ubiquitin–proteasome system
miRNAs	MicroRNAs
AGO	Argonaute
RISC	RNA-induced silencing complex
CDP-DAG	cytidine diphosphate glycerol
PtdInsP2	phospholipid inositol diphosphate
BIC	biotype interacting complex
EIHM	Extra-invasive hyphal membrane
TPR	tetratricopeptide repeats
IAA	auxin
GA	gibberellins
SOD	Superoxide Dismutase
AOX	aldehyde oxidase
CAT	Catalase
DAMP	damage-associated molecular patterns
GWAS	Genome-wide association analysis
MAS	Marker-Assisted Selection
GS	Genomic Selection
HR	hypersensitive response
PCD	programmed cell death
CRISPR/Cas9	Clustered Regularly Interspaced Short Palindromic Repeats

## Appendix A

**Table A1:** Summary of the main *R* genes of cloned rice blast in rice.

Gene Name	Coding Protein	Chr	Cloning Method	Donor	Reference
*Pit*	CNL	1	Map-based cloning	K59	[135]
*Pish* ^a^	NLR	1	Mutagenesis-based cloning	Nipponbarc	[136]
*Pi35* ^a^	NLR	1	Map-based cloning	Hokkai-188	[137]
*Pi37*	NLR	1	Map-based cloning	St. No. 1	[138]
*Pi64*	CNL	1	Map-based cloning	Yangmaogu	[39]
*Pib* ^b^	NLR	2	Map-based cloning	BL1	[28,139]
*rbr2* ^b^	CNL	2	Map-based cloning	Minghui 63	[140]
*Pi63*	CNL	4	Map-based cloning	Kahei	[141]
*Pid2*	B-lectin RLK	6	Map-based cloning	Digu	[30]
*Pid3* ^d^	NLR	6	Comparative Genomics	MC276	[142]
*pid4* ^d^	CNL	6	Transcriptome and comparative genomics	Digu	[143]
*Pid3-A4* ^d^	NLR	6	Allelic mining	A4 (*Oryza rufipogon*)	[144]
*Pi25* ^d^	NLR	6	Map-based cloning	Gumei2	[145]
*Pi9* ^c^	CNL	6	Map-based cloning	75-1-127	[27]
*Pi2* ^c^	CNL	6	Map-based cloning	C101A51	[146]
*Piz-t* ^c^	CNL	6	Map-based cloning	Toride 1	[146]
*Pi50* ^c^	CNL	6	Map-based cloning	Er-Ba-zhan	[147]
*Pigm* ^c^	CNL	6	Map-based cloning	Gumei 4	[148]
*Pizh* ^c^	CNL	6	Map-based cloning	Zhonghua 11	[149]
*Pi36*	CNL	8	Map-based cloning	Q61	[150]
*Pi5* ^e^	CNL	9	Map-based cloning	RIL260	[151]
*Pii* ^e^	CNL	9	Allele mining	Fujisaka5	[36]
*Pik* ^h^/*Pi54* ^g^	CNL	11	Map-based cloning	Tetep	[152]
*Pi54rh* ^g^	CNL	11	Allele mining	Oryza rhizomatis	[153]
*Pi54of* ^g^	CNL	11	Allele mining	Oryza officinalis	[154]
*Pia*	NLR	11	Multifaceted genomics approach	Nipponbare	[155]
*Pik* ^f^	NLR	11	Map-based cloning	Kusabue	[156]
*Pike* ^f^	CNL	11	Map-based cloning	Xiangzao 143	[157]
*Piks* ^f^	CNL	11	Allele mining	IRBLKs-F5	[26]
*Pi1* ^f^	CNL	11	Map-based cloning	C101LAC	[158]
*Pik-p* ^f^	CNL	11	Map-based cloning	K60	[159]
*Pik-m* ^f^	CNL	11	Map-based cloning	Tsuyuake	[160]
*Pik-h* ^f^	CNL	11	Map-based cloning	K3	[161]
*Pikg* ^f^	CNL	11	Allele mining	accession G9	[162]
*Pi65*	LRR-RLK	11	Map-based cloning	GangYu65	[24]
*Pik-W25* ^f^	CNL	11	BSA mapping	WR25	[108]
*Pi-ta*	NLR	12	Map-based cloning	Katy	[163]
*Ptr*	ARM repeats	12	Map-based cloning	BHA	[33]

^a−h^ These marked genes are allelic to each other.

**Table A2:** Information on rice blast-resistant varieties.

S. No	Variety	Breeding Techniques	Researcher or Unit
1	Chunjiang 25	Crossbreeding	Wu Mingguo et al. [164]
2	shangyou 63	Crossbreeding	Xiuxia Yang et al. [165]
3	Tiejing 16	MAS	Ma et al. [166]
4	K59	Crossbreeding	Yang Wangxing et al. [167]
5	Nipponbarc	Crossbreeding	Aichi Prefectural Agricultural Experiment Station [168]
6	Hokkai-188	Crossbreeding	Nguyen, Fukuoka et al. [138]
7	St. No. 1	Genome Editing	Hayano-Saito [169]
8	Yangmaogu	Crossbreeding	Jian Ma et al. [39]
9	BL1	Backcross	Sedeek, S et al. [170]
10	Minghui 63	Crossbreeding	Wang et al. [171]
11	Kahei	Mas	Miyamoto M., Yano M., Hirasawa H et al. [141]
12	Digu	Crossbreeding	Lihuang Zhu, Xuewei Chen et al. [30]
13	MC276	Gs	Wan, Moroberekan et al. [172]
14	Gumei2	Crossbreeding	Jie Chen [145]
15	Tetep	Mas	Atul Singh [173]
16	Zhonghua 11	Gs	Xue-Feng Yao [174]
17	Q61	Crossbreeding	Xie et al. [150]
18	Fujisaka5	Backcross	Fujimaki Hiroshi et al. [175]
19	Oryza rhizomatis	Crossbreeding	Vaughan et al. [154]
20	Nipponbare	Crossbreeding	Okuyama et al. [155]
21	Kusabue	Crossbreeding	Zhai et al. [156]
22	Xiangzao 143	Gs	Hunan Rice Research Institute [157]
23	K60	Crossbreeding	Yuan et al. [159]
24	YF47	Backcross	Mao et al. [106]

**Table A3:** Information on races of rice blast strains [176,177].

Source Cultivarsa	Year of Isolation	Races	State	Frequency of Occurrence
Caffey	2014	IC-17	Arkansas	The most common race
CL 151	2013	1B-25	Arkansas	Uncommon race
HR12		MG01	south of indian	The most common race
CL 151	2015	1B-1	Arkansas	The most common race
CL 261	2013	IA-37	Arkansas	Uncommon race
70-15		MG-8	south of indian	The most common race
CL 261	2013	IA-69	Arkansas	Uncommon race
Jupiter	2015	IA-113	Arkansas	Uncommon race
CL 261	2012	1B-17	Arkansas	The most common race
Jupiter	2013	IG-1	Arkansas	Uncommon race
CL 261	2012	1B-49	Arkansas	The most common race
Jupiter	2015	IA-1	Arkansas	Uncommon race
CL 261	2012	IC-9	Arkansas	Uncommon race
CL 261	2013	IE-1	Arkansas	The most common race
		FJ81278	fujian province china	The most common race
CL 262	2013	1B-41	Arkansas	Uncommon race
Colorado	2013	IC-1	Texas	Uncommon race
CL 261	2013	IA-65	Arkansas	Uncommon race
Jupiter	2013	1B-21	Louisiana	Uncommon race
LA 2025	2013	1B-37	Louisiana	Uncommon race
		HN19311	hunan province in china	The most common race
